# Origin and role of hepatic myofibroblasts in hepatocellular carcinoma

**DOI:** 10.18632/oncotarget.27532

**Published:** 2020-03-31

**Authors:** Betul Gok Yavuz, Roberto Carmagnani Pestana, Yehia I. Abugabal, Sunil Krishnan, Jian Chen, Manal M. Hassan, Robert A. Wolff, Asif Rashid, Hesham M. Amin, Ahmed O. Kaseb

**Affiliations:** ^1^Department of Basic Oncology, Hacettepe University Cancer Institute, Ankara, Turkey; ^2^Department of Gastrointestinal Medical Oncology, The University of Texas MD Anderson Cancer Center, Houston, TX, USA; ^3^Department of Radiation Oncology, The University of Texas MD Anderson Cancer Center, Houston, TX, USA; ^4^Department of Gastroenterology, Hepatology, and Nutrition, The University of Texas MD Anderson Cancer Center, Houston, TX, USA; ^5^Department of Epidemiology, The University of Texas MD Anderson Cancer Center, Houston, TX, USA; ^6^Department of Pathology, The University of Texas MD Anderson Cancer Center, Houston, TX, USA; ^7^Department of Hematopathology, The University of Texas MD Anderson Cancer Center, Houston, TX, USA; ^8^MD Anderson Cancer Center UTHealth Graduate School of Biomedical Sciences, Houston, TX, USA

**Keywords:** hepatocellular carcinoma, stellate cells, myofibroblasts, liver fibrosis, immunotherapy

## Abstract

Hepatocellular carcinoma (HCC) is the most common primary liver cancer and is the second leading cause of cancer-related death worldwide. Fibrosis and cirrhosis are important risk factors for the development of HCC. Hepatic myofibroblasts are the cells responsible for extracellular matrix deposition, which is the hallmark of liver fibrosis. It is believed that myofibroblasts are predominantly derived from hepatic stellate cells (HSCs), also known as Ito cells. Nevertheless, depending on the nature of insult to the liver, it is thought that myofibroblasts may also originate from a variety of other cell types such as the portal fibroblasts (PFs), fibrocytes, hepatocytes, hepatic progenitor cells (HPCs), and mesothelial cells. Liver myofibroblasts are believed to transform into cancer-associated fibroblasts (CAFs) while HCC is developing. There is substantial evidence suggesting that activated HSCs (aHSCs)/cancer-associated fibroblasts (CAFs) may play an important role in HCC initiation and progression. In this paper, we aim to review current literature on cellular origins of myofibroblasts with a focus on hepatitis B virus (HBV)- and hepatitis C virus (HCV)-induced hepatic fibrosis. We also address the role of aHSCs/CAFs in HCC progression through the regulation of immune cells as well as mechanisms of evolvement of drug resistance.

## INTRODUCTION

HCC is the most common primary liver cancer and is the second leading cause of cancer-related death worldwide [1]. Globally, the incidence of HCC is increasing, including in the United States, with chronic hepatitis C virus (HCV) and non-alcoholic steatohepatitis (NASH) are considered the most prevalent risk factors [2]. In addition, there are several other risk factors that predispose to HCC including heavy alcohol intake and certain metabolic diseases such as hemochromatosis and alpha-1-antitrypsin deficiency. Geographical variations in HCC etiology are best exemplified by the fact that the most common etiology of liver cirrhosis, a prominent pre-HCC clinicopathological condition, is HCV in the United States and Egypt, in contrast to HBV in Asia and some other developing countries [3].

Fibrosis is characterized by abundant extracellular matrix deposition, especially collagen I, as a result of persistent inflammation and continuous injury to organs such as the liver, kidney, and lungs [4]. HCC is a special type of cancer because 80–90% of the cases develop in a background of advanced liver fibrosis and cirrhosis [5]. Liver cirrhosis is one of the most serious complications of chronic liver diseases, with 5-10% of patients with liver cirrhosis developing HCC within 5 years [5]. Notably, majority of HCC patients experience fibrosis and cirrhosis before cancer development in contrast to most other types of cancer where fibrosis typically occurs as a reactive process in response to the tumor [6].

Historically, fibrosis was considered an irreversible process until recently, where several animal studies have shown that reversal of fibrosis may occur when the liver insult is removed [7]. Furthermore, evolving clinical evidence also confirms regression of hepatic fibrosis when the triggering insult is ceased. For example, in patients with chronic HBV infection, long-term suppression of viral replication with tenofovir disoproxil fumarate resulted in regression of fibrosis and even cirrhosis [8]. In one study of 38 patients with HCV, 61 months after sustained virologic response (SVR) following interferon and ribavirin, 61% of the responders had regression of cirrhosis and 89% demonstrated decreased liver collagen content [9]. Notably, although fibrosis reversal prevented patients from developing complications of hepatic cirrhosis, it did not eliminate the risk of HCC development in spite of reducing such risk [10, 11]. Unlike viral hepatitis-driven liver fibrosis, studies showing clinical evidence of regression of fibrosis in patients with alcoholic or non-alcoholic liver disease are lacking [7].

## CELLULAR ORIGIN OF HEPATIC MYOFIBROBLASTS

In a healthy liver, extracellular matrix turnover is precisely regulated by an intricate balancing act between a group of enzymes called matrix metalloproteinases (MMPs) in one side, and their inhibitors, tissue inhibitors of metalloproteinases (TIMPs) on the other side [12]. Upon damage of the liver due to chronic inflammation, the expression of TIMPs and collagens is upregulated in activated fibroblasts. This results in inhibition of MMPs and subsequent deposition of matrix proteins especially collagen type 1, which subsequently causes up to a tenfold increase in fibrotic matrix [13].

**Figure 1:**
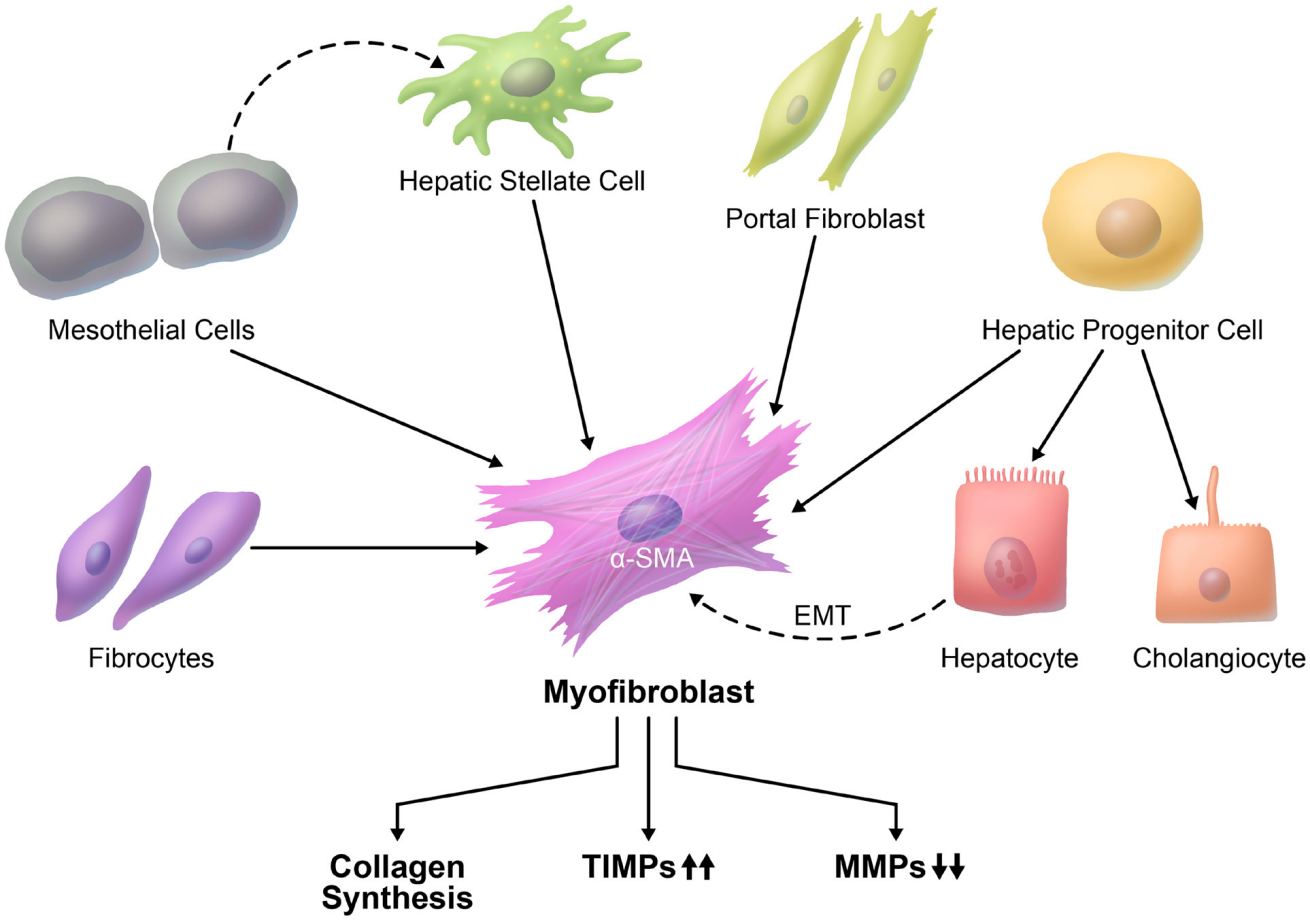
Cellular origin of liver fibrosis. There is more than one proposed origin of myofibroblasts in liver fibrosis. A major source of myofibroblasts is hepatic stellate cells. Myofibroblasts can also originate from portal fibroblasts, hepatic progenitor cells, mesothelial cells, and circulating fibrocytes. It is not clear whether myofibroblasts can be derived from hepatocytes through EMT (dashed line).

The main cell type that is responsible for fibrosis development is the myofibroblast, which is absent in normal liver [14]. Myofibroblasts are characterized by the expression of α-smooth muscle actin (α-SMA), collagen type I. Beginning at the earliest stages of hepatocellular carcinogenesis and coincident with the development of HCC, the myofibroblasts transform into cancer-associated fibroblasts (CAFs) [6]. Despite the extensive studies in this field, the exact cellular origin of liver myofibroblasts is largely unknown. Depending on the nature of injury to the liver, the origin of liver myofibroblasts may vary considerably [15] (Table 1). In fact, there is a strong evidence that hepatic stellate cells (HSCs) constitute a major fibrogenic cell population that is responsible for liver fibrosis (Figure 1). It is essential to elucidate the cell of origin of myofibroblasts, the mechanisms and signaling pathways that contribute to transformation of myofibroblasts, and crosstalk with cancer cells that leads to progression of HCC.

**Table 1:** Potential cellular origin of hepatic myofibroblasts

Potential Cell Types	Potential Biomarkers	References
Hepatic stellate cells (HSCs)	• GFAP • Desmin • LRAT • PDGFRβ • HAND2	[17, 18]
Portal fibroblasts (PFs)	• Elastin • Thy-1 • NTPDase2	[15, 24]
Hepatocytes	• FSP-1 • FAP	[28, 32]
Hepatic progenitor cell (HPCs)	• CD133 • CK-7	[34, 35]
Fibrocytes	• CD11b, CD34, CD45, CD54, CD80, CD86, CCR1, CCR2, CCR5, CCR7, MHCII, Gr1, Ly6c • Collagen type 1, Fibronectin, Vimentin	[36, 38]
Mesothelial cells (MCs)	• GPM6 • ITGA8	[45, 46]

Abbreviations: GFAP: glial fibrillary acidic protein. LRAT: lecithin retinol acyltransferase. PDGFRβ: platelet-derived growth factor receptor-β. HAND2: heart- and neural crest derivatives-expressed protein 2. NTPDase2: Ectonucleoside Triphosphate Diphosphohydrolase 2. FSP-1: fibroblast specific protein-1. FAP: Fibroblast Activation Protein Alpha. CK-7: Type II Cytoskeletal 7. MHC II: Major Histocompatibility Complex, Class II. GPM6: Glycoprotein M6. ITGA8: integrin α8.

### Hepatic stellate cells

HSCs are located in between the hepatocytes and the sinusoidal endothelial cells (space of Disse), and constitute about 10% of all resident liver cells [16]. In the normal liver, HSCs are characterized by a non-proliferative, quiescent phenotype. Vitamin A lipid droplets in their cytoplasm represent the most distinctive feature of quiescent HSCs and provide an easy way to isolate these cells by means of density gradient centrifugation and flow cytometry [17]. There are also other markers that help to distinguish HSCs from other hepatic cells: glial fibrillary acidic protein (GFAP), desmin, lecithin retinol acyltransferase (LRAT), platelet-derived growth factor receptor-β (PDGFRβ), and heart- and neural crest derivatives-expressed protein 2 (HAND2) [18].

HSCs are activated during chronic liver injury and, during this process, HSCs gradually lose their lipid droplets and become myofibroblasts as regard to their secretory and phenotypic profile [18]. Activated HSCs are considered a major source of collagen deposition in liver fibrosis [19]. According to a study in transgenic mice, 82-96% of myofibroblasts originated from HSCs independent of the type of liver injury [20, 21]. Thus, HSCs stand out as precursors to myofibroblasts and as a primary cellular target for preventing or treating liver fibrosis prior to HCC development [21].

Although no direct relationship between liver fibrosis and hepatocellular carcinogenesis has been conclusively established, there is substantial evidence suggesting that HSCs may play a role in HCC initiation and progression [6]. Myofibroblasts in HCC is widely thought to be originated from HSCs [6]. An important study by Zhou et al. has shown that HSCs are activated by HCC cell-derived exosomes in a concentration-dependent manner and they are transformed α-SMA and FAP positive CAFs [22]. These activated HSCs further promote cancer progression by secreting angiogenic cytokines [22]. In another study, a 12-marker panel of CAFs in HCC has identified from the Cancer Genome Atlas (TCGA) database, which is associated with both pathological and clinical progressions of cancer [23]. To validate the panel, HSC cell line LX2 is cultured with conditioned medium of Huh7 HCC cell line and cultured LX2 cells show increased expression of 12 marker proteins in addition to typical CAFs markers, α-SMA and FAP [23].

### Portal fibroblasts (PFs)

PFs are quiescent spindle-shaped fibroblasts that are located in the peri-portal space. Because of the paucity of markers identifying PFs, it is hard to distinguish these cells from other mesenchymal cells [15]. Elastin, Thy-1, and NTPDase2 are classically seen on PFs but not on HSCs [24]. Whereas the contribution of activated PFs to liver fibrosis is important during early stages of cholestatic liver injury, it has been shown that activated HSCs constitute the major source of myofibroblasts during late stages of cholestatic liver injury and in hepatotoxic liver injury [20].

### Hepatocytes

Epithelial-mesenchymal transition (EMT) is a process characterized by epithelial cells gaining motility and mesenchymal features [25]. EMT plays important roles in tumor cell invasion and metastasis [26]. In addition, EMT potentially plays a key role in fibrogenesis. This is best characterized in the fibrotic kidney where 36% of interstitial fibroblasts originate from tubular epithelial cells via EMT [25, 27]. Interestingly, Zeisberg et al. have shown that hepatocytes may contribute to the population of FSP-1 (fibroblast specific protein-1)-positive cells by undergoing EMT in CCl_4_-induced liver fibrosis [28]. FSP1 is expressed not only in fibroblasts but also in leukocytes and other non-fibroblastic cell types [29]. Meanwhile, there are several lineage tracing animal studies that have shown strong evidence against this claim. For example, Taure et al. utilized triple transgenic mice to demonstrate that hepatocytes do not undergo EMT to transform to fibrotic myofibroblasts *in vivo* [30]. Similarly, another study indicated that EMT of cholangiocytes traced by genetic labeling does not contribute to hepatic fibrosis in mice [31].

Myofibroblasts in HCC may be originated from HCC cells undergoing EMT. Hypoxia induces increased expression FAP, which is a typical marker for CAFs, in HCC cells through HIF-1α [32]. In another study, it is shown that TGF-β, which regulates EMT and fibroblast maturation, induces α-SMA in HCC cells [33]. However, it is unclear that whether this phenotypical change in HCC cells is correlated with functional features of CAFs and further studies are needed to clarify this issue.

### Hepatic progenitor cell (HPCs)

HPCs are bipotent progenitor cells that give rise to hepatocytes and cholangiocytes in response to liver injury [34]. In a recent study, Sekiya *et al.* have shown that HPCs isolated from mice injured liver can differentiate into myofibroblasts, as a third distinct cell type, in addition to hepatocyte and cholangiocyte differentiation. They also have proposed that HPCs can contribute to the formation of the premalignant niche by abundant production of myofibroblasts [35].

### Fibrocytes

Fibrocytes are bone marrow-derived cells that express both hematopoietic (CD11b, CD34, CD45, CD54, CD80, CD86, CCR1, CCR2, CCR5, CCR7, MHCII, Gr1, and Ly6c) as well as mesenchymal (collagen type 1, fibronectin, and vimentin) markers [36]. In response to TGF-β, fibrocytes differentiate into α-SMA-positive myofibroblasts. This event is associated with decreased expression of hematopoietic markers [37]. After recruitment to the site of injury, fibrocytes differentiate into myofibroblasts [38].

Only a small number of fibrocytes can be detected in the peripheral blood from healthy individuals [39]. Nonetheless, the number of circulating fibrocytes is increased under pathological conditions such as pulmonary fibrosis, which suggests that circulating fibrocytes may serve as a biomarker of disease progression [39–41]. In contrast, studies based on genetic lineage tracing mouse models of liver fibrosis have shown that myofibroblasts are mostly derived from sources other than the bone marrow suggesting that the contribution of fibrocytes to liver fibrosis is negligible [20, 21].

### Mesothelial cells (MCs)

MCs are simple squamous cells lining the surface of visceral organs and body cavities (peritoneum, pericardium, and pleura). They secrete lubricating fluid to decrease the friction during organ movement. MCs exhibit both epithelial and mesenchymal features [42]. Studies have shown that MCs can migrate into the liver and give rise to HSCs in mouse liver development [42, 43]. A recent study using a mouse model has demonstrated that Wilms tumor 1 (Wt-1) expressing MCs can give rise to HSCs and myofibroblasts during liver fibrosis via mesothelial-mesenchymal transition [44]. On the contrary, in a study based on the bile duct ligation mouse model, cells of injured liver tissue have been isolated by flow cytometric sorting for GPM6, a mesothelial cell marker, GPM6-positive cells did not express type 1 collagen mRNA, suggesting that mesothelial cells may not always transform into myofibroblasts during chronic liver injury [45]. Interestingly, a recent article reported that HSCs may show different gene expression profiles depending on their cell of origin [46]. In mice, the cell lineage tracing of Wt-1-expressing MCs revealed that, upon differentiation from MCs, MC-derived HSCs lose the expression of MC markers and gain the expression of integrin α8 (ITGA8), making ITGA8 a unique cell surface marker for MC-derived HSCs in the developing liver [46]. Collectively, these data suggest that MCs may contribute to liver fibrosis indirectly through differentiation to HSCs. However, further studies are warranted to clarify the relative contribution of mesothelial cells to HSCs.

## PATHOPHYSIOLOGICAL MECHANISMS OF LIVER FIBROSIS

Since HSCs are the main cell type contributing to liver fibrosis, most published literature is focused on HSC activation as the main triggering factor leading to liver fibrogenesis. The activation of HSCs encompasses a complex process that includes cellular and molecular interactions [18]. Briefly, initial liver injury by various etiologies such as viral hepatitis causes hepatocyte death resulting in inflammatory cell recruitment to the injury site. Thereafter, reactive oxygen species production and cytokine secretion (especially TGF-β) from inflammatory cells or hepatocytes activate specific intracellular pathways within HSCs. This leads to changes in HSCs behavior including stimulation of chemotaxis, proliferation, contractility, and ultimately fibrogenesis [47].

**Figure 2:**
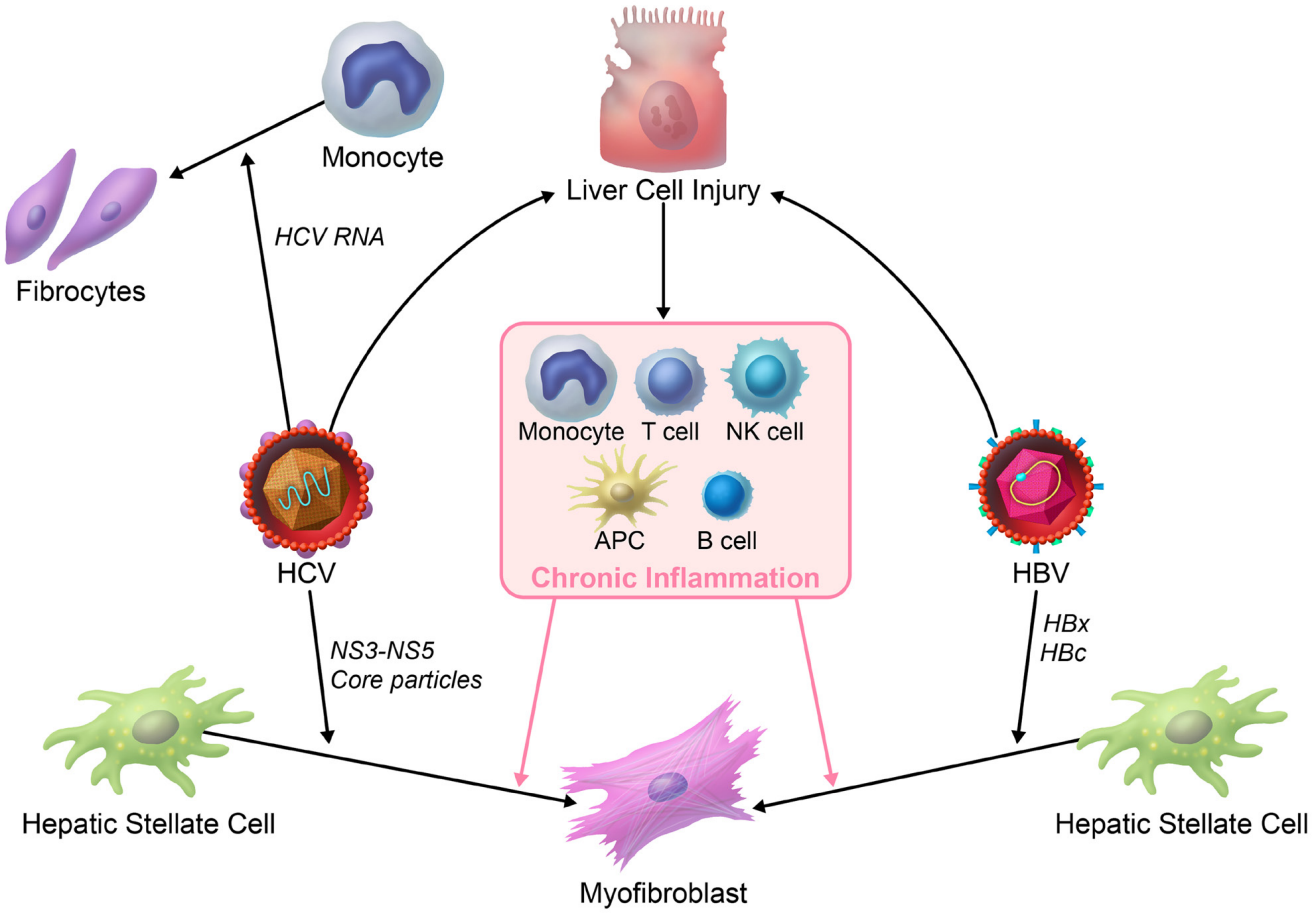
HBV- vs. *HCV-induced liver fibrosis*. HBV- or HCV-infected hepatocytes and/or inflammation activates hepatic stellate cells through soluble factors. There are some direct acting mechanisms that may lead to liver fibrosis in viral hepatitis. HCV virus can activate hepatic stellate cells by NS3-NS5 and core particles. It is also illustrated that HCV RNA can differentiate monocytes to fibrocytes. HBV HBx and HBc particles are also believed to activate hepatic stellate cells.

Different etiologies can induce liver fibrosis, but whether the course by which the fibrosis develops is modulated by etiology-specific factors, such as HBV vs. HCV is not clear, mainly because of the lack of pertinent animal models. In this review we specifically focus on the direct effects of HCV and HBV on liver fibrosis mostly based on *in vitro* experiments (Figure 2).

### HCV-mediated liver fibrosis

HCV is a single-stranded RNA virus that can translate its polyprotein precursor in host cells. The precursor protein gives rise to structural (core, E1, E2, and p7) and non-structural (NS2, NS3, NS4A, NS4B, NS5A, and NS5B) proteins via proteolytic cleavage [48]. These proteins can act as key cellular regulators in an autocrine or paracrine manner [49]. In fact, core proteins can easily be detected in the serum of chronic HCV patients [50]. Core proteins of HCV can directly activate HSCs by interacting with C1q to increase proliferation of HSCs. On the other hand, NS3 and NS5 can activate NF-κB and c-Jun N-terminal kinase pathways of HSCs and increase the expression of collagen-I in HSCs [51]. HCV envelope protein 2 (E2) can also increase the expression of MMP-2 in HSCs through E2/CD81 interaction [52].

HCV can directly infect human skin fibroblasts and induce the expression of pro-fibrotic proteins such as vimentin, collagen, and α-SMA by activating GLI2 (glioma-associated oncogene family zinc finger 2), which has been previously linked to organ fibrosis [53, 54]. Since HSCs are considerably different from skin fibroblasts [55], this finding should also be confirmed in HSCs.

Fibrocytes are another cell type that may contribute to HCV-mediated liver fibrosis. Patients who are infected with HCV have higher levels of circulating fibrocytes in their peripheral blood than healthy people [56]. Although the exact mechanisms of fibrocyte-induced fibrosis in HCV-infected patients is not fully understood, it has been recently shown that, during HCV infection, viral ssRNA differentiates monocytes to collagen-expressing fibrocytes via TLR 7/TLR 8 [57]. Further studies are needed to elucidate how these fibrocytes are recruited to the injury site and whether they may serve as a biomarker of disease progression like in patients with pulmonary fibrosis.

### HBV-mediated liver fibrosis

The HBV has a partially double-stranded DNA encoding the C, S and X proteins, reverse transcriptase, and polymerase [58]. Although the role of these viral proteins on tumor development has been extensively studied, their possible role on fibrogenesis is a relatively new subject in the literature. Bai et al., have shown that after co-culture with the cells expressing HBV C and X viral proteins or incubation with HBV particles, HSC cell line LX-2 cells proliferate and increase their expression of α-SMA and collagen I [59, 60]. Similarly, the expression of collagen I, α-SMA, and TIMP-1 is increased in HSC-T6 cells (rat HSCs) after transfection of the hepatitis B envelope antigen (HBeAg) [61]. These effects are mediated by increased TGF-β secretion in HSC-T6 cells and neutralization of TGF-β can prevent their activation [61]. Unlike the HBV X and HBV C proteins, endogenous expression of HBV pre-core protein does not induce fibrogenic changes in HSCs [62].

### Macrophages in liver fibrosis

Macrophages constitute the largest part of the non-parenchymal cellular compartment of the liver and important for liver fibrosis [63]. There are different types of macrophages in the liver in terms of origin and function: Tissue-resident macrophages, Kupffer cells (KC) or bone marrow-monocyte derived macrophages (MoMFs) [63]. KCs are particularly important during homeostasis such as iron metabolism and clearance of systemic pathogens, whereas MoMFs are recruited to the liver tissue when liver damage occurs and have an active role in liver inflammation [64].

KCs get activated following the liver injury and secrete many chemotactic stimuli (e. g. CCL2) for inflammatory cells. This results in the infiltration of a large number of MoMFs into the liver. These macrophages secrete pro-inflammatory cytokines and chemokines resulting in the activation of HSCs to fibrogenic phenotype [65].

Liver macrophages perform diverse functions in different stages of liver fibrosis. Impaired monocyte recruitment in liver results in reduced HSC activation and liver fibrosis [65, 66]. However it is also shown that liver macrophages can exert fibrinolytic functions by secreting MMPs and anti-inflammatory cytokines especially at later stages of liver fibrogenesis [67, 68]. Liver macrophages show high plasticity that can change their phenotype and function according to stimulus they receive from their environment, which can explain their opposing functions in liver fibrogenesis [69].

As liver macrophages have important functions in liver fibrosis and are abundant in the liver, they have been seen as a potential target for treating liver fibrosis [70]. Potential strategies targeting macrophages include preventing the infiltration of monocytes, antagonizing the pro-inflammatory cytokines they secrete, modifying the functional activation status of macrophages to promote the liver fibrinolysis [71–73].

## ACTIVATED HSCS/CAFS, HCC DRUG RESISTANCE AND HCC IMMUNOTHERAPY

Tumors are believed as ‘’wounds that not heals’’ as chronic inflammation and fibrosis is generally precedes with tumor initiation. Several types of cells in the tumor microenvironment (immune cells, endothelial cells, and fibroblasts, etc.) support this process [74, 75]. Fibrosis is particularly important for HCC development as 80–90% of the cases develops in a background of advanced liver fibrosis and cirrhosis [5]. That’s why, HSCs which are major fibrogenic cells in liver fibrosis are believed to transform into cancer associated fibroblasts in HCC and, CAFs are loosely defined as HSCs in HCC tissue [6, 22].

Activated HSCs signature is associated with earlier recurrence of HCC after resection [76]. HSCs may promote hepatocellular carcinogenesis in many ways [6]. For instance, activated HSCs induce angiogenesis and proliferation of tumor cells, as well as maintain the survival of tumor cells directly through mediators released from fibroblasts or indirectly via interactions with other cells. Herein, we specifically focus on the role of activated HSCs and CAFs on immunotherapy and drug resistance in HCC, which are emerging subjects and not reviewed elsewhere.

**Figure 3:**
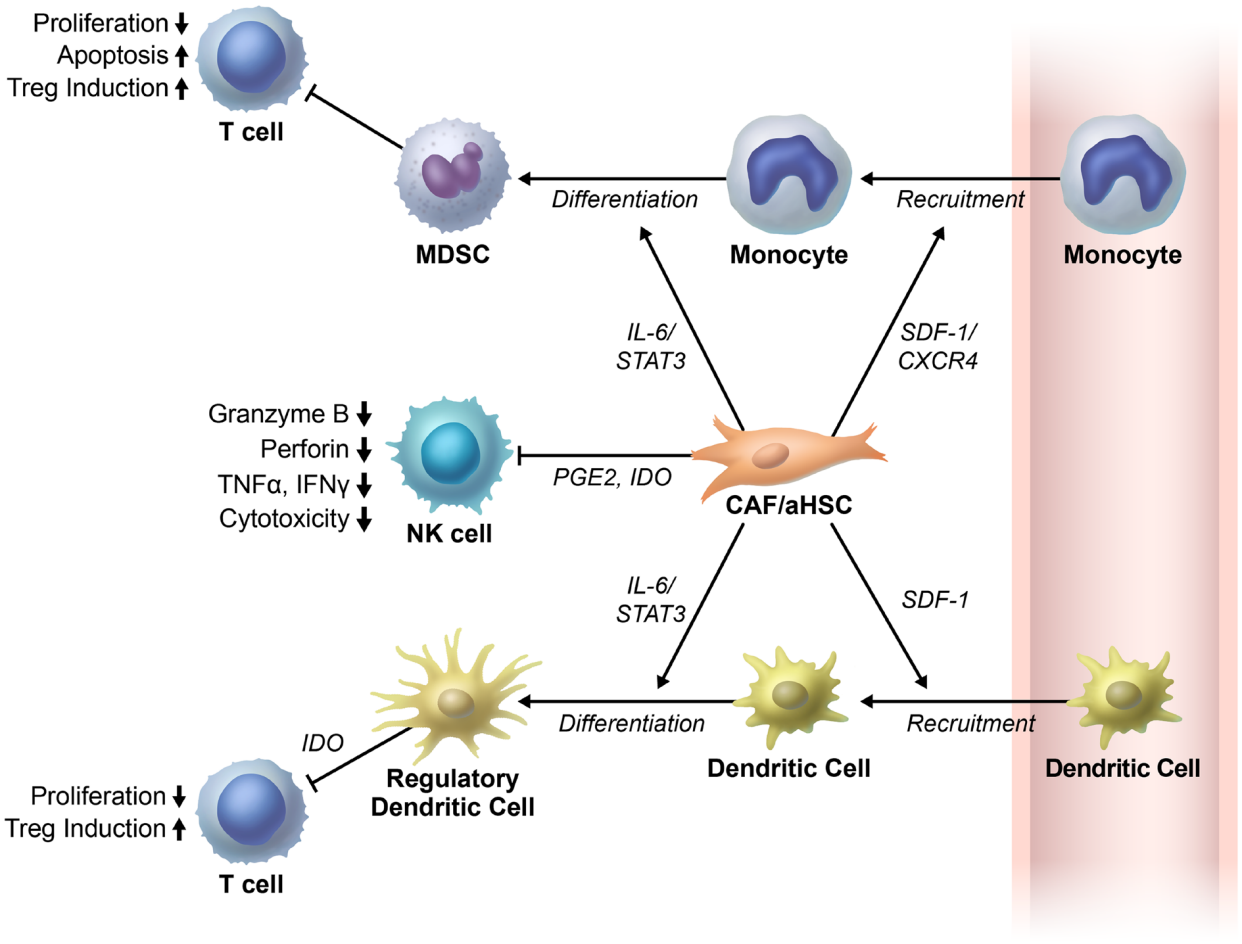
Cancer associated fibroblasts/activated hepatic stellate cells regulate immune cells in tumor microenvironment. Cancer associated fibroblasts/activated hepatic stellate cells (CAFs/aHSCs) inhibit T-cell proliferation directly (not shown in figure) or indirectly. They first recruit the monocytes and dendritic cells. Thereafter, CAFs/aHSCs differentiate the monocytes and dendritic cells to myeloid derived suppressor cells (MDSCs) and regulatory dendritic cells, respectively. Subsequently, these cells exhibit immunosuppressive functions in the tumor microenvironments. Moreover, the cancer associated fibroblasts (CAFs) in the liver impair NK cell functions. The NK cells play important roles in the anti-tumor immune response through prostaglandin E2 (PGE2) and indoleamine 2,3-dioxygenase (IDO). Abbreviations: SDF, stromal cell-derived factor.

There is a continuous crosstalk between stromal cells such as fibroblasts and immune cells in tumor microenvironment [77]. This crosstalk plays an important role in the development and progression of tumors [77]. Liver activated fibroblasts have recently gained significant interest for their potential function as regulators of immune cell recruitment and function (Figure 3).

A study by Zhao et al., has shown that HSCs inhibit T-cell proliferation. Co-transplantation of HSCs and H22 (murine hepatic cancer cell line) results in less immune cell infiltration in the tumor tissue, more regulatory T cells and myeloid derived suppressor cells (MDSCs) in the spleen and bone marrow, and more apoptotic mononuclear cells in the tumor tissue than transplantation of H22 cells alone in orthotopic liver tumor mouse models [78, 79].

Deng et al., have shown that tumor associated fibroblasts (TAFs) isolated from HCC tissues recruit monocytes via the stromal cell-derived factor (SDF)-1/CXCR4 pathway and differentiate them into myeloid-derived suppressor cells (MDSCs) through the IL-6/STAT3 axis. Furthermore, in human HCC tissue samples, CD11-positive myeloid cells, which resemble TAF-educated monocytes are found to have a positive association with TAFs [80].

Regulatory dendritic cells (DCs) exhibit an immune suppressive phenotype by inhibiting T-cell proliferation through indoleamine 2,3-dioxygenase (IDO) production in tumor tissues [81]. Cheng et al., have shown that HCC-associated fibroblasts can recruit normal DCs and transform them to regulatory DCs through IL-6-mediated STAT3 activation. Moreover, inhibition of IL-6 and STAT3 can reverse this effect [82].

Natural killer (NK) cells are another important innate immune cell type that kill tumor cells. Fibroblasts isolated from human HCC tissue disrupt NK cell functions. NK cells co-cultured with HCC fibroblasts have shown decreased expression of cytotoxic molecules and surface markers for cell activation through prostaglandin E2 (PGE2) and IDO [83]. HSCs-derived PGE2 has also been shown to play a role in MDSCs recruitment to the tumor tissues. Moreover, inhibition of HSCs-derived PGE2 results in inhibition of HSCs-induced MDSCs accumulation in tumor tissue and thus suppression of HCC growth [84].

Identification of oncogenic addiction pathways in different tumor types has led to revolutionary therapeutic approaches in many cancers, such as imatinib for treatment of chronic myeloid leukemia [85]. Unlike many other cancer types, HCC does not show an addiction to a specific survival pathway. Moreover, tumor microenvironment may modify the sensitivity of tumor cells to inhibition of oncogenic pathways in HCC [86]. Recent studies have shown that HSCs may play a role in drug resistance. The HSC cell line LX2 cocultured with the HCC cells Huh7, can induce sorafenib resistance in these cells through multiple survival pathways including HGF/c-Met/Akt and JAK2/STAT3. The addition of S3I-201 (phosphorylated-STAT3 inhibitor) to the co-culture medium enhances sorafenib-induced apoptosis [87]. In another study, HSCs secreted extracellular matrix proteins and HSCs conditioned medium decreased the effectiveness of sorafenib on HCC cells via restoration of FAK activity through abrogation of ubiquitination [88]. It has also been reported that HSCs in multicellular tumor spheroids (MCTS) exhibited pronounced resistance to sorafenib and cisplatin compared to other types of stromal cells. Moreover, the therapeutic efficacy of sorafenib is increased in MCTS by using losartan which suppresses collagen I synthesis by HSCs [89].

Interestingly, some recent studies challenge the idea that stromal cells merely exert pro-tumorigenic functions. For example, targeting CAFs in mouse models of pancreatic ductal adenocarcinoma enhances tumor growth instead of inhibiting it [90]. Similarly, increased expression of the orphan receptor endosialin (CD248) in HSCs is inversely correlated with tumor cell proliferation in human HCC tumors, suggesting that this protein may be a negative regulator of HCC progression [91]. Further studies are needed to further characterize the mechanisms of switching between pro-tumorigenic and anti-tumorigenic effects of HSCs.

## PROGNOSTIC AND DIAGNOSTIC CYTOKINE BIOMARKERS IN HCC PATIENTS WITH CHRONIC VIRAL HEPATITIS AND LIVER FIBROSIS

Biomarkers found in serum, tissue or other body fluids are important tools for screening, prognosis, prediction, and monitoring response to therapy in cancer. α-fetoprotein (AFP) is the most widely used diagnostic and prognostic serum biomarker in HCC, but most small HCC do not secrete AFP. In fact, AFP sensitivity drops to 25% if the tumor is smaller than 3 cm in diameter [92]. Therefore, new biomarkers are required to enhance routine HCC diagnostic and prognostic schemes and improve early detection. Furthermore, such new biomarkers are expected to lead to identifying more effective therapeutic options that will ultimately improve survival of HCC patients. Several serum or tissue biomarkers have been recently proposed. Alas, most of these biomarkers have not been widely accepted in daily clinical practice [93, 94].

Persisting chronic liver injury induces a continuous cycle of inflammation, regeneration and proliferation of hepatocytes that results in accumulation of pathophysiologic aberrancies that lead to fibrosis, cirrhosis, and HCC initiation, as discussed above in detail [95]. However, 20% of patients with HBV develop HCC without evident cirrhosis [96]. This may be partly explained by the fact that HBV can integrate its own DNA into the host genome resulting in disruption of critical regulatory pathways and initiation of carcinogenesis [97, 98]. Unlike HBV, HCV (RNA virus) cannot integrate its own genome into the host DNA. Therefore, HCV-induced HCC mostly develops in the context of liver cirrhosis suggesting that an indirect pathway of chronic inflammation may have a role in the carcinogenesis process in HCV-associated HCC [99].

Entrenched within this framework of viral infection and transformation of resident liver cells into fibrogenic cells and/or cells with genomic instability is an environment that is permissive for initiation and progression of HCC. A key component of this permissive environment is the crosstalk between resident cells, migratory cells, and freshly recruited cells that is mediated by cytokines, which are important molecules for cellular interactions and especially in mediating the immune response to alterations in integrity of the liver structure and physiology. Since inflammation is a major response to viral hepatitis and possibly fibrosis and HCC development, cytokines in the liver and in the blood maybe used as a biomarker for disease progression. For example, Hsia et al. showed that the serum levels of IL-6 and IL-10 are frequently elevated in patients with HCC [100]. In the same study, among patients with low AFP levels, increased IL-6 or IL-10 levels were significantly associated with the presence of HCC suggesting that these cytokines may be used as a tumor marker for patients with low AFP [100]. These cytokines have also ben related to liver fibrosis, interestingly, and their role in the diagnosis of the continuum from liver disease to HCC deserves further studies [101, 102]. Although extensive research is ongoing in this field, the knowledge about the use of cytokines as diagnostic and prognostic tumor biomarkers in HCC is limited. Considering the difference in the mechanisms by which HBV and HCV can cause HCC and fibrosis, serum levels of possible prognostic and diagnostic biomarkers in patients with HBV or HCV should be evaluated separately. Table 2 shows possible serum cytokine biomarkers that are differentially expressed in HCC patients compared to patients with chronic viral hepatitis stratified by HBV and HCV status.

**Table 2:** Potential cytokine biomarkers in HCC patients with chronic viral hepatitis

Cytokine	Sample size	Regulation	Information	Reference
**HBV-related cytokines**				
Resistin	*n* = 33 (HBV-HCC)	up	Differentiate HCC from CHB	[99]
	*n* = 120 (CHB)			
IL-7	*n* = 33 (HBV-HCC)	down	Differentiate HCC from CHB	[99]
	*n* = 120 (CHB)			
IL-8	*n* = 33 (HBV-HCC)	up	Differentiate HCC from CHB	[99]
	*n* = 120 (CHB)			
MDC	*n* = 43 (HBV-HCC)	down	Differentiate HCC from CHB	[104]
	*n* = 33 (CHB)			
MSPα	*n* = 43 (HBV-HCC)	down	Differentiate HCC from CHB	[104]
	*n* = 33 (CHB)			
IL-6	*n* = 153 (HBV-LC)	up	Differentiate HCC from HBV-LC	[105]
	*n* = 148 (HBV-HCC)		Differentiate HCC from CHB	
	*n* = 37	up	Differentiate HCC from CHB	[106]
**HCV-related cytokines**				
TNF-α	*n* = 10 (HCV-HCC)	up	Differentiate HCC from CHC	[107]
	*n* = 10 (HCV)			
MIG	*n* = 10 (HCV-HCC)	up	Differentiate HCC from CHC	[107]
	*n* = 10 (HCV)			
TRAIL	*n* = 10 (HCV-HCC)	up	Differentiate HCC from CHC	[107]
	*n* = 10 (HCV)			
APRIL	*n* = 10 (HCV-HCC)	up	Differentiate HCC from CHC	[107]
	*n* = 10 (HCV)			
VEGF	*n* = 10 (HCV-HCC)	up	Differentiate HCC from CHC	[107]
	*n* = 10 (HCV)			
IL-3	*n* = 10 (HCV-HCC)	up	Differentiate HCC from CHC	[107]
	*n* = 10 (HCV)			
TWEAK	*n* = 10 (HCV-HCC)	up	Differentiate HCC from CHC	[107]
	*n* = 10 (HCV)			
SCF	*n* = 10 (HCV-HCC)	up	Differentiate HCC from CHC	[107]
	*n* = 10 (HCV)			
IL-21	*n* = 10 (HCV-HCC)	up	Differentiate HCC from CHC	[107]
	*n* = 10 (HCV)			
FGF2	*n* = 10 (HCV-HCC)	up	Differentiate HCC from CHC	[107]
	*n* = 10 (HCV)			
GRO-alpha	*n* = 10 (HCV-HCC)	up	Differentiate HCC from CHC	[107]
	*n* = 10 (HCV)			
Exotaxin-3	*n* = 10 (HCV-HCC)	up	Differentiate HCC from CHC	[107]
	*n* = 10 (HCV)			
IL-22	*n* = 10 (HCV-HCC)	up	Differentiate HCC from CHC	[107]
	*n* = 10 (HCV)			
TNF-αR2	*n* = 20 (HCV-HCC)	up	Differentiate HCC from HCV-LC	[108]
	*n* = 28 (HCV-LC)		Differentiate HCC from CHC	
	*n* = 25 (CHC)			
IFN-γ	*n* = 20 (HCV-HCC)	down	Differentiate HCC from HCV-LC	[108]
	*n* = 28 (HCV-LC)		Differentiate HCC from CHC	
	*n* = 25 (CHC)			
IL-1β	*n* = 20 (HCV-HCC)	up	Differentiate HCC from HCV-LC	[108]
	*n* = 28 (HCV-LC)		Differentiate HCC from CHC	
	*n* = 25 (CHC)		
IL-10	*n* = 20 (HCV-HCC)	up	Differentiate HCC from HCV-LC	[108]
	*n* = 28 (HCV-LC)		Differentiate HCC from CHC	
	*n* = 25 (CHC)			
	*n* = 20 (HCV-HCC)	up	Differentiate HCC from HCV-LC	[109]
	*n* = 20 (HCV-LC)		Differentiate HCC from HCV	
	*n* = 20 (HCV)			
IL-2	*n* = 20 (HCV-HCC)	down	Differentiate HCC from HCV-LC	[108]
	*n* = 28 (HCV-LC)		Differentiate HCC from CHC	
	*n* = 25 (CHC)			
IL-6	*n* = 120	up	Differentiate HCC from CHC	[110]
	*n* = 20 (HCV-HCC)	up	Differentiate HCC from HCV-LC	[109]
	*n* = 20 (HCV-LC)		Differentiate HCC from HCV	
	*n* = 20 (HCV)			
	*n* = 20 (HCV-HCC)	up	Differentiate HCC from HCV-LC	[111]
	*n* = 20 (HCV-LC)		Differentiate HCC from HCV	
	*n* = 20 (HCV)			
IL-17	*n* = 20 (HCV-HCC)	up	Differentiate HCC from HCV-LC	[111]
	*n* = 20 (HCV-LC)		Differentiate HCC from HCV	
	*n* = 20 (HCV)			
sTNF-RII	*n* = 30 (HCV-HCC)	up	Differentiate HCC from CHC	[112]
	*n* = 32 (CHC)		Differentiate HCC from CHC-PNALT	
	*n* = 17 (CHC-PNALT)			
sIL-2Ra	*n* = 30 (HCV-HCC)	up	Differentiate HCC from PNALT	[112]
	*n* = 32 (CHC)			
	*n* = 17 (CHC-PNALT)			
IL-8	*n* = 30 (HCV-HCC)	up	Differentiate HCC from CHC	[112]
	*n* = 32 (CHC)		Differentiate HCC from CHC-PNALT	
	*n* = 17 (CHC-PNALT)			

Abbreviations: CHB, chronic hepatitis B; CHC, chronic hepatitis C; HBV, hepatitis B virus; HCC, hepatocellular carcinoma; HCV, hepatitis C virus; LC, liver cirrhosis PNALT, persistent normal alanine aminotransferase.

## CONCLUSIONS

This review provides a summary of recent literature on the pathogenesis of liver fibrosis that is often a prelude to HCC development in the setting of chronic viral hepatitis. A preponderance of studies suggest that HSCs are precursors to myofibroblasts, the cells responsible for ECM deposition that is the hallmark of fibrosis. Nevertheless, there are other possible routes to myofibroblast development and recruitment that implicate PFs, hepatocytes, fibrocytes, and MCs. It also appears that the exact cell of origin of HSCs is possibly being dictated by the nature of liver injury. Furthermore, the molecular signaling pathways that transform these cells, HSCs in particular, into mediators of fibrogenesis are varied and highly context dependent. Convergence of multiple factors related to direct transformation of cells into cancer cells, paracrine effects of other stromal cells, and immune system alterations contribute to hepatocellular carcinogenesis and treatment resistance within the microenvironment of chronic liver injury. Relevant observations addressed in this review carry significant importance considering recent emergence of immune modulatory-based therapies as a legitimate approach to treat HCC [103].

## Abbreviations

α-SMA: alpha-smooth muscle actin
AFP: alpha fetoprotein
aHSC: activated hepatic stellate cells
CAFs: cancer associated fibroblasts
CCl_4_: carbon tetrachloride
CHB: chronic hepatitis B
CHC: chronic hepatitis C
DC: dendritic cells
E2: envelope protein 2
ECM: extracellular matrix
EMT: epithelial-mesenchymal transition
FSP-1: fibroblast specific protein-1
GFAP: glial fibrillary acidic protein
GLI2: glioma-associated oncogene family zinc finger 2
HAND2: heart- and neural crest derivatives-expressed protein 2
HBeAg: hepatitis B envelope antigen
HBV: hepatitis B virus
HCC: hepatocellular carcinoma
HCV: hepatitis C virus
HPC: hepatic progenitor cell
IDO: indoleamine 2,3-dioxygenase
ITGA8: integrin α8
LC: Liver cirrhosis
LRAT: lecithin retinol acyltransferase
MC: mesothelial cell
MDSC: myeloid derived suppressor cells
MMP: matrix metalloproteinases
NASH: non-alcoholic steatohepatitis
NK cell: natural killer cell
PDGFRβ: platelet-derived growth factor receptor-β
PF: portal fibroblasts
PGE2: prostaglandin E2
PNALT: persistent normal alanine aminotransferase
SDF-1: stromal cell-derived factor-1
SVR: sustained virologic response
TAF: tumor associated fibroblasts
TCGA: the Cancer Genome Atlas
TIMP: tissue inhibitors of metalloproteinase
Wt-1: Wilms tumor-1
